# *Cordyceps militaris* Grown on Germinated Soybean Suppresses KRAS-Driven Colorectal Cancer by Inhibiting the RAS/ERK Pathway

**DOI:** 10.3390/nu11010020

**Published:** 2018-12-21

**Authors:** HeeJung Seo, Jisu Song, Minyoung Kim, Dong-Wook Han, Hye-Jin Park, Minjung Song

**Affiliations:** 1 Department of Food Biotechnology, Division of Bioindustry, Silla University, Busan 46958, Korea; ssk4623@naver.com (H.S.); jisu632@naver.com (J.S.); 2 Department of Cogno-Mechatronics Engineeering, College of Nanoscience & Nanotechnology, Pusan National University, Busan 46241, Korea; nanohan@pusan.ac.kr; 3 Department of Medical Science, School of Medicine, Pusan National University, Yangsan 50612, Korea; 4 Department of Electronics & IT Media Engineering, Seoul National University of Science & Technology, Seoul 01811, Korea; mkim21@gmail.com; 5 Department of Food Biotechnology, Gachon University, Kyungji-do 13120, Korea; nimpi79@daum.net

**Keywords:** *Cordyceps militaris*, colorectal cancer, SW480, KRAS

## Abstract

*Cordyceps militaris* is a commonly used medicinal mushroom containing various therapeutic effects such as anti-inflammatory, anti-allergic, and anti-cancer activities. This study examined whether *Cordyceps militaris* on germinated soybeans (GSC) has a suppressive effect on a v-ki-ras2 Kirsten rat sarcoma viral oncogene homolog (KRAS)-driven colorectal cancer which is notorious for its un-druggable features and the ineffectiveness of conventional therapies against it. GSC extract was prepared and its proximate composition and amino acids were analyzed. The suppressive effects were investigated with the KRAS-driven colorectal cancer cell-line, SW480. SW480 proliferation, clonogenic potential, apoptosis, and the RAS/extracellular signal-regulated kinase (ERK) pathway under the GSC treatment were analyzed by 3-(4,5-dimethylthiazol-2-yl)-5-(3-carboxymethoxyphenyl)-2-(4-sulfophenyl)-2H-tetrazolium (MTS) assay, flow cytometry, and Western blot, respectively. An *in vivo* experiment with the SW480 xenograft mouse model was performed. As a result, GSC suppressed cell proliferation by inducing the apoptosis of KRAS-driven colorectal cancer cells and inhibited clonogenic capabilities. The decrease of KRAS and ERK phosphorylation was detected by Western blot. Tumor growth was significantly suppressed when GSC was introduced to the tumor-xenograft mouse model. In conclusion, GSC suppressed KRAS-driven colorectal cancer growth both in vitro and in vivo, and can be used as an alternative or simultaneous approach in colorectal cancer therapy.

## 1. Introduction

Colorectal cancer (CRC) is the third most prevalent cancer worldwide [1]. Approximately 1.7 million new cases were diagnosed in 2016 and 830,000 people were estimated to have died as a result [1]. In general, the incidence of CRC is high in developed countries compared to developing countries, but the cases are increasing worldwide due to nutritional transitions. The increasing prevalence has prompted extensive research into the development of therapies. On the other hand, 30% to 40% of CRC patients have a v-ki-ras2 Kirsten rat sarcoma viral oncogene homolog (KRAS)-driven CRC, which is difficult to target with drugs due to its notorious ‘un-druggable’ property and ineffective response to conventional therapies [2]. RAS proteins are the most powerful cancer drivers. KRAS-driven colorectal cancer is initiated from the epidermal growth factor (EGF) binding to the EGF receptor (EGFR), which activates GTP-bound KRAS. The activated KRAS continuously transmits a signal to the downstream RAS-ERK cell regulation pathways. The ERK proteins activated by their phosphorylation are translocated to the nucleus and activate the transcription factors that involve the cell cycle progression and transcription. Hence, they are involved directly in tumor progression. The abnormal levels of phosphorylated mitogen-activated protein kinases (MAPKs), ERK1, and ERK2 are observed in ~30% of human tumors [2,3]. To inhibit RAS activation, targeting EGFR with monoclonal antibodies such as Cetuximab has emerged as a potential therapeutic [4,5]. However, monoclonal antibody therapeutics have side effects and a second resistance harboring KRAS mutation, which accounts for 30% to 40% of CRC patients [6,7,8,9].

There is evidence that natural products exhibit anti-colorectal cancer activities. For example, Frondoside A isolated from a sea cucumber enhanced the anti-cancer effects of colon cancer drugs [10]. Imperatorin isolated from *Angelica dahurica* suppressed the proliferation and angiogenesis of colorectal cancer by targeting hypoxia-inducible factor (HIF)-1α [11]. Han et al. reported that Ginsenoside 20 –Rh2 from *Panax ginseng* inhibited colon cancer cell proliferation through the JAK2/STAT3 pathway [12]. However, there are few natural products reported to act against KRAS-driven colorectal cancer. 

*Cordyceps militaris* is a functional mushroom that has been used traditionally to treat inflammatory diseases [13] and improve the immune system [14,15]. Several reports have demonstrated its suppressive activity on cancer progression [16,17]. On the other hand, naturally occurring *Cordyceps militaris* has limited availability because of its high production cost. Park et al. inoculated *Cordyceps militaris* onto germinated soybeans (*Glycine max (L.) Merr*) (GSC) to improve its productivity and studied its functions [18,19,20]. In their functional study, GSC exhibited therapeutic activity on colon inflammatory disease [18], allergic diseases [19,20], and cancer [21]. Considering the information above, the extracts of GSC containing chemical compounds may modulate the proliferation and progression of mCRC.

This study examined the anti-cancer activity of GSC on G12V (mutation occurring at codon 12)–KRAS driven colorectal cancer. The amino acid composition was analyzed. The proliferation profile and clonogenic features of the KRAS-driven cell line, SW480, were evaluated under the GSC treatment. The RAS/MEK/ERK and AKT/PI3K pathways on cancer cell death were investigated. In addition, GSC extracts were administered to colon cancer cell xenograft mice to examine its anti-cancer effects in vivo. 

## 2. Materials and Methods

### 2.1. Preparation of Cordyceps militaris Grown on Germinated Soybeans Extract

The *Cordyceps militaris* (Kucari 0906) grown on germinated soybeans (GSC) was provided by the Cell Activation Research Institute (CARI, Seoul, Korea). Authenticated voucher specimens of GSC were deposited in the Herbarium at the Department of Food Science and Biotechnology, Gachon University (Seongnam, Gyeonggido, Korea). GSC was ground to a fine powder with a grinder and reconstituted with 80% ethanol for 48 h. The solution was dried with a rotary evaporator under vacuum and stored at −20 °C until needed. GSC was then dissolved in distilled water.

### 2.2. Proximate Composition and Analysis of Amino Acids

The GSC sample was analyzed for its crude protein, crude fat, and crude ash contents. The crude protein content was determined based on the total nitrogen content and the Kjeldahl method. The crude fat was extracted with petroleum ether by the Soxhlet method (AOAC, 2000) [22]. The contents of all the amino acids were determined by using an amino acid analyzer (Biochrom 30^+^, Biochrom, Cambridge, UK).

### 2.3. Cell Viability by the MTS Proliferation Assay and DAPI/PI Staining

The G12V KRAS-mutated SW480, G12D KRAS-mutated LS513, G13D KRAS-mutated HCT116, KRAS-WT/v-raf murine sarcoma viral oncogene homolog B1 (BRAF)-mutated HT-29, KRAS-WT/BRAF-WT Caco-2 CRCs and KRAS-WT/BRAF-WT A549 lung cancer cells were purchased from the American Type Culture Collection (Rockville, MD, USA). Cells were maintained in RPMI-1640 or DMEM (Invitrogen, Carlsbad, CA, USA) with 10% fetal bovine serum (FBS, Invitrogen) and 100 U/mL penicillin–streptomycin (Sigma, St. Louis, MO, USA). The media was changed every 2 to 3 days with regular passage 1 to 2 times a week. Before cell seeding, cells were detached from the flasks with 0.25 % (*v*/*v*) trypsins and 0.02% Ethylene diamine tetraacetic acid (EDTA) solution. For the cell viability assay, the cells were seeded onto 96-well plates (20,000 cells/well) and treated with an increasing concentration of GSC (1–500 μg/mL) after 24 h. For proliferation analysis, 3-(4,5-dimethylthiazol-2-yl)-5-(3-carboxymethoxyphenyl)-2-(4-sulfophenyl)-2H-tetrazolium (MTS) kit (Promega, Fitchburg, WA, USA) was used, according to the manufacturer’s instructions. More specifically, the MTS reagent was added on the media for 1 hour at 37 °C and absorbance readings were taken at 490 nm (Model 550, Bio-Rad, Hercules, CA, USA).

For 4′,6-diamidino-2-phenylindole (DAPI)/ propidium iodide (PI) staining, the cells were fixed with 4% paraformaldehyde (Sigma). The cells were stained with 1 μg/mL of 4′,6-diamidino-2-phenylindole (DAPI, Sigma) for 10 min at room temperature, which was followed by propidium iodide (Sigma) incubation in the dark. The stained cells were then observed under an inverted fluorescence microscope (EVOS^®^, Thermo Fisher Scientific, Waltham, MA, USA).

### 2.4. Clonogenic Assay

For a clonogenic assay, the cells were seeded onto a 6-well plate (1000 cells/well) [23]. After one day, the cells were treated with GSC extracts in different concentrations (1, 10, 100, and 500 µg/mL) and Cetuximab (30 µg/mL). After the 10–day cultivation, the cells were stained with a 0.05% crystal violet (Sigma) for 20 min at room temperature. The experiments were performed in triplicate.

### 2.5. Flow Cytometry

The extent of SW480 apoptosis after GSC treatment was evaluated by using an Annexin V-fluorescein isothiocyanate (FITC)/PI apoptosis detection kit (BD Biosciences, San Jose, CA, USA), according to the manufacturer’s instructions. SW480 colorectal cancer cells were seeded overnight in a 6-well plate (5 × 10^5^ cells/well) and treated with Cetuximab (30 μg/mL) and the GSC extract (1–500 μg/mL). After 24 h, the cells were harvested, washed in ice-cold PBS, and collected by centrifugation at 500 g for 10 min. The cells were stained simultaneously with FITC-labeled annexin V (5 µL) and PI (5 µL) at room temperature for 10 min and protected from light. The stained cells were analyzed by using a fluorescence-activated cell sorter flow cytometer (FC 500 Series Flow Cytometry, Beckman Coulter, Indianapolis, IN, USA). At least 10,000 cells were used for each analysis and the experiments were conducted in triplicate.

### 2.6. Western Blotting

The cells were seeded in 6-well plates with a density of (5 × 10^5^ cells/well) and treated with GSC (1–500 µg/mL) for 48 h. The cells were detached, washed in cold PBS, and re-suspended in RIPA buffer containing the protease inhibitors (Sigma). The suspension was kept on ice for 20 min and then centrifuged at 15,000× *g* for 30 min at 4 °C. The proteins (20 μg) were loaded onto each lane, separated on 10% pre-cast SDS-PAGE gel (Invitrogen), and followed by transfer in the iBlot system (Thermo Scientific). The membranes were blocked with a bovine serum albumin solution (Sigma) for 1 h at room temperature and incubated with the following primary antibodies. The antibodies for KRAS (1:1000), phospho-ERK1/2 (1:2000), ERK1/2 (1:1000), AKT (1:1000), phospho-AKT (1:1000) and HSP90 (1:1000) were obtained from Cell Signaling Technology (Danvers, MA). The antibody for the β-actin (1:1000) was obtained from Sigma. The membranes were then incubated with horseradish peroxidase-conjugated goat anti-rabbit or anti-mouse antibodies for 2 h (Pierce, Rockford, IL, USA). The blots were detected by using a SuperSignal^®^ West Femto enhancer kit (Pierce). Densitometry analysis was performed by using ImageJ (NIH, Bethesda, MD, USA). The ratios of KRAS/β-actin, phospho-pERK/ERK, and phospho-AKT/AKT were calculated with the control representing 1.0-fold changes, which is shown below. 

### 2.7. Tumor Growth Analysis In Vivo

The inhibitory effects of GSC on colorectal cancer growth were investigated in an animal model. Twenty-one 6-week-old BALB/c athymic nude mice (male) were obtained from the BioToxTech animal center (Cheongju, Korea). Animal care and handling procedures were performed in compliance with the experimental protocol approved by the Institutional Animal Care and Use Committee (IACUC) at Silla University (Busan, Korea). SW480 cells were harvested from the culture plate and re-suspended in PBS. A total of 5 × 10^6^ cells in 0.1 mL of media were injected subcutaneously into both sides of nude mice [24]. When engrafted, the tumor volume was measured daily with calipers and calculated by using the following formula: V = (L × W^2^)/2, where V = volume, L = length, and W = width. When the tumors reached approximately 100 mm^3^ in size, the mice were assigned randomly to their respective treatments (Cetuximab, GSC extract, and saline control, *n* = 7/group). The mice were treated twice per week with an intraperitoneal injection of 10 mg/kg Cetuximab in PBS. The GSC extracts were administered by an oral gavage (400 mg/kg/day) every day for 23 days. 

### 2.8. Statistical Analysis 

The data are expressed as the means ± standard error of the mean (SEM). Statistical analyses were performed by one-way analysis of variance (ANOVA) using SPSS software, version 12 (SPSS Inc., Chicago, IL, USA). The differences were considered significant at *p* < 0.05.

## 3. Results

### 3.1. Proximate Composition and Analysis of Amino acids 

GSC consisted of 55% crude protein, 13% crude ash, and less than 1% crude fat (Table 1). Table 2 lists the profiles of the amino acids in GSC. A total of 18 amino acids were analyzed. GSC contained all the essential amino acids (EAA), which are isoleucine (Ile), leucine (Leu), lysine (Lys), methionine (Met), phenylalanine (Phe), threonine (Thr), valine (Val), and tryptophan (Trp). Leucine and threonine were found at high levels. Non-essential amino acids (NEAA) of cysteine (Cys), aspartic acid (Asp), glycine (Gly), glutamic acid (Glu), alanine (Ala), serine (Ser), proline (Pro), and tyrosine (Tyr) were also found in GSC, with glutamic acid being the most abundant. Based on amino acid analysis, GSC contained high levels of delicious amino acids (DAA) including Glu, Asp, Gly, and Ala. 

### 3.2. GSC Suppressed KRAS-Mutated Colorectal Cancer Cell Proliferation

**Figure 1:**
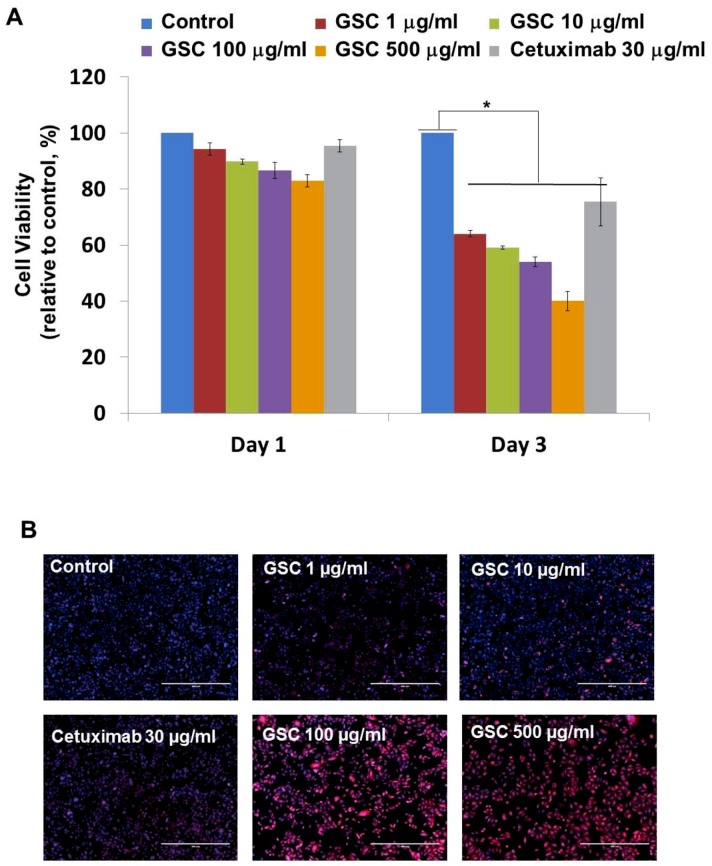

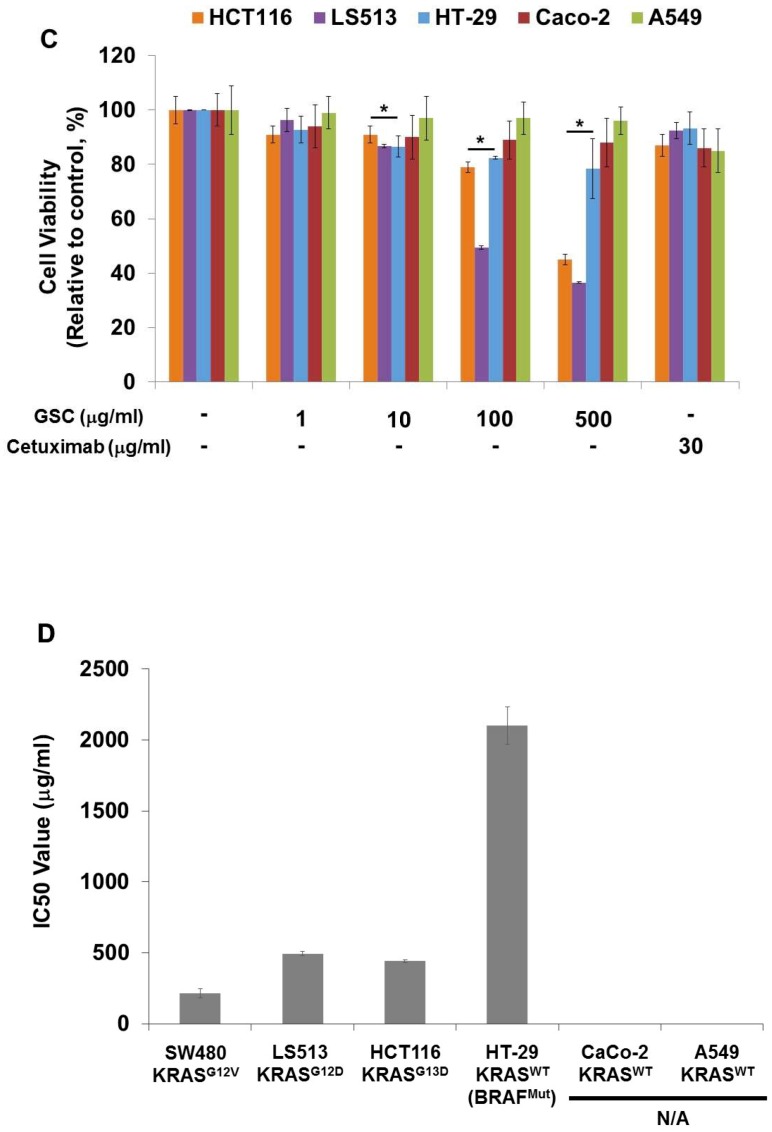
(**A**) Cell viability of SW480 colorectal cancer cells at days 1 and 3 with the GSC treatment (0, 1, 10, 100, and 500 μg/mL). The relative cell viability was compared to the control. The data are expressed as the mean ± standard error of the mean (SEM) and a significant difference is denoted as * (*p* < 0.05). (**B**) Representative SW480 cell images after the GSC treatment (0, 1, 10, 100 and 500 μg/mL). Blue represents the DAPI-stained cell nuclei and the propidium iodide-stained dead cells are red. (Scale bar = 500 μm). (**C**) Cell viability of LS513 (Kirsten rat sarcoma viral oncogene homolog (KRAS)^G12D^), HCT116 (KRAS^G13D^), HT-29 (KRAS^WT^/BRAF^Mut^), Caco-2 (KRAS^WT^/BRAF^WT^) and A549 (KRAS^WT^/BRAF^WT^) during day 3 with GSC (0, 1, 10, 100, and 500 μg/mL) and Cetuximab (30 μg/mL) treatment. The relative cell viability was compared with the control. (**D**) IC_50_ values of SW480 (KRAS^G12V^), LS513 (KRAS^G12D^), HCT116 (KRAS^G13D^), HT-29 (KRAS^WT^/BRAF^Mut^), Caco-2 (KRAS^WT^/BRAF^WT^) and A549 (KRAS^WT^/BRAF^WT^) cell lines at day 3 under GSC treatment. The data are expressed as the mean standard error of the mean (SEM) and a significant difference is denoted as * (*p* < 0.05) compared to control. GSC: *Cordyceps militaris* on germinated soybeans. DAPI, 4′,6-diamidino-2-phenylindole.

SW480, which is the KRAS^G12V^-mutated colorectal cancer cell line, was chosen to evaluate the GSC antitumor potential. The cells were treated with GSC at different concentrations and Cetuximab as the drug control. Figure 1A presents SW480 colorectal cancer cell proliferation in the presence of GSC extracts at different concentrations (0, 1, 10, 100, and 500 μg/mL) on day 1 and 3. The cell growth was calculated as the relative percentage absorbance compared to the control. As a result, treating SW480 colon cancer cells with GSC suppressed cell proliferation in a dose-dependent manner when compared to the control. For example, the cell viability on day 3 was decreased by up to 54.2 ± 2.95% with the 100 µg/mL GSC treatment and 40.0 ± 2.2% with the 500 µg/mL GSC treatment. This led to the selection of doses for further mechanistic and molecular studies, which ranged from 1 to 500 μg/mL. For morphological observations, the cells were stained with PI and DAPI under the GSC treatment (Figure 1B). Dead cells were stained with PI and were expressed as a red color. The cell nuclei from the dead and live cells were stained as DAPI (blue color). As shown in Figure 1B, most cells were only stained with blue DAPI in the control group, which showed that most cells were alive after 48 h of cultivation. On the other hand, the PI-stained dead cells began to appear and their ratios increased with a rise in the GSC concentration. In addition, the GSC extract fraction suppressed the proliferation on different types of KRAS mutations, e.g., LS513 (KRAS^G12D^), HCT116 (KRAS^G13D^), HT-29 (KRAS^WT^/BRAF^Mut^), Caco-2 (KRAS^WT^/BRAF^WT^) and A549 (KRAS^WT^/BRAF^WT^) (Figure 1C). In the KRAS^WT^/BRAF^Mut^ HT-29 CRC line, the GSC extract also inhibited the proliferation, but had less effect on the proliferation at the same concentration ranges (1, 10, 100, and 500 µg/ mL) (Figure 1C). As seen in Figure 1C, GSC did not show a suppressive effect on KRAS^WT^/BRAF^WT^ Caco-2 CRC and A549 lung cancer cell proliferation. The IC_50_ values on KRAS mutated CRC cell lines were 214 μg/mL (SW480), 493 μg/mL (LS513) and 441 μg/mL (HCT116) (Figure 1D). IC_50_ of HT-29 increased up to approximately 10-fold and IC_50_ of KRAS^WT^/BRAF^WT^ cells of Caco-2 and A549 was not applicable due to its minimal effect. Figure 1 demonstrated that KRAS-mutated CRC cell lines showed more sensitive responses under GSC treatment than the response of the KRAS wild-type cell line.

### 3.3. GSC Extract Alters Clonogenic Characteristics

**Figure 2:**
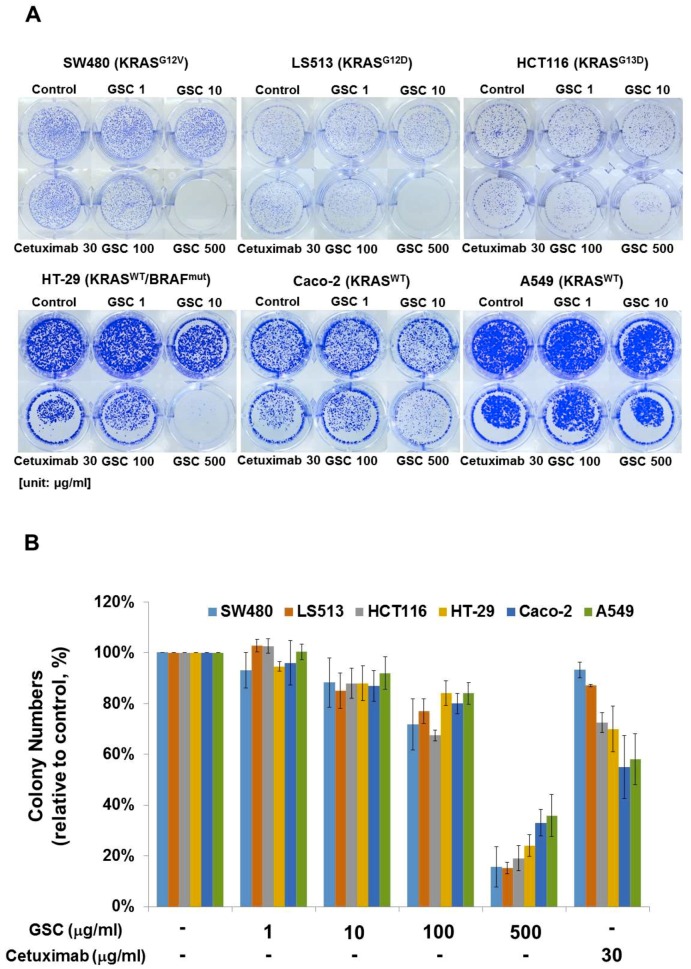
(**A**) Clonogenic potential after the GSC extract treatment (0, 1, 10, 100, and 500 μg/mL) on day 10 on SW480 (KRAS^G12V^), LS513 (KRAS^G12D^), HCT116 (KRAS^G13D^), HT-29 (KRAS^WT^/BRAF^Mut^), Caco-2 (KRAS^WT^/BRAF^WT^) and A549 (KRAS^WT^/BRAF^WT^). (**B**) Relative colony numbers upon the GSC treatment on SW480 (KRAS^G12V^), LS513 (KRAS^G12D^), HCT116 (KRAS^G13D^), HT-29 (KRAS^WT^/BRAF^Mut^), Caco-2 (KRAS^WT^/BRAF^WT^) and A549 (KRAS^WT^/BRAF^WT^). The data are expressed as the mean ± standard error of the mean (SEM). GSC: *Cordyceps militaris* on germinated soybeans.

The clonogenic potential was evaluated to determine the long-term anti-proliferative activity of GSC on colorectal cancer cells. The colony-forming ability is one of the tumor cell characteristics and is tightly correlated with tumorigenes in vivo. As shown in Figure 2A, the SW480 colonies disappeared or could not be formed at the highest GSC concentration. A similar tendency was also observed on other KRAS mutant cells known as LS513 (KRAS^G12D^) and HCT116 (KRAS^G13D^). The clonogenic potential of SW480 was suppressed significantly by GSC in a dose-dependent manner when compared to the controls (Figure 2B). For example, the colony numbers were reduced by up to 72 ± 10% and 16 ± 8% after 100 and 500 µg/mL GSC treatment, respectively. In particular, the reductions in the colony number were accompanied by reductions of the colony size. Under Cetuximab treatment, the colony numbers were 93 ± 3%, which might indicate a cell resistant property to the drug in long-term incubation. In case of KRAS wild-type cells, the clonogenic potential was suppressed by GSC treatment. However, this was relatively low compared to the KRAS-mutated group. These findings suggest that the treatment of colorectal cancer cells with GSC would decrease their rate of proliferation and their tumor-formation ability.

### 3.4. GSC Extract-Induced KRAS-Driven Colorectal Cancer Cell Apoptosis 

**Figure 3:**
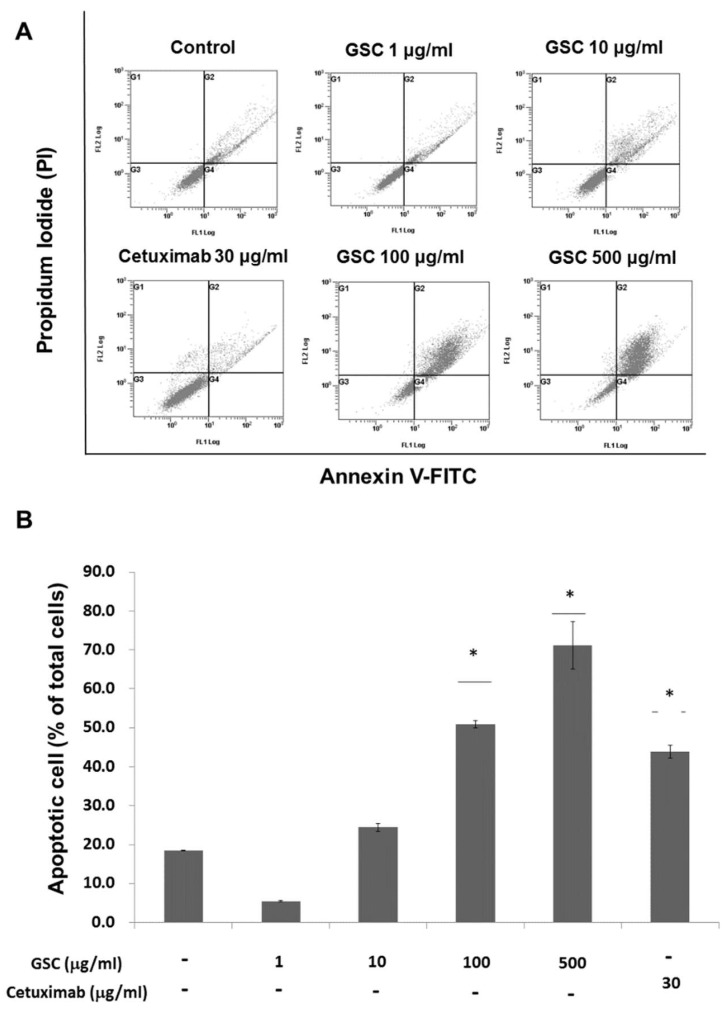
(**A**) Flow cytometry plots using Annexin V-FITC/PI staining for apoptosis. SW480 cells were treated for 24 h and then stained with Annexin V-FITC/PI upon GSC treatment (0, 1, 10, 100, and 500 μg/mL). (**B**) The percentage of apoptotic cells upon the GSC treatment were compared statistically and the significant differences are denoted by * (*p* < 0.05). GSC: *Cordyceps militaris* on germinated soybeans, FITC, fluorescein isothiocyanate, PI, propidium iodide. The data are expressed as the mean ± standard error of the mean (SEM).

Annexin V-FITC/PI staining and flow cytometry were performed to examine GSC effects on cell death (Figure 3). Flow cytometry (FACS) analysis indicated that the GSC extract induced apoptosis in a concentration-dependent manner. For example, the GSC treatment (10 μg/mL) induced early (annexin V FITC^+^/PI^-^) and late (annexin V FITC^+^/PI^+^) apoptosis of 20.9 ± 0.23%. The apoptotic rate increased to 56.3 ± 6.07% and 72.3 ± 1.64% with 100 and 500 μg/mL, respectively.

### 3.5. Effects of GSC Extract on the RAS/ERK Signaling Pathways 

**Figure 4:**
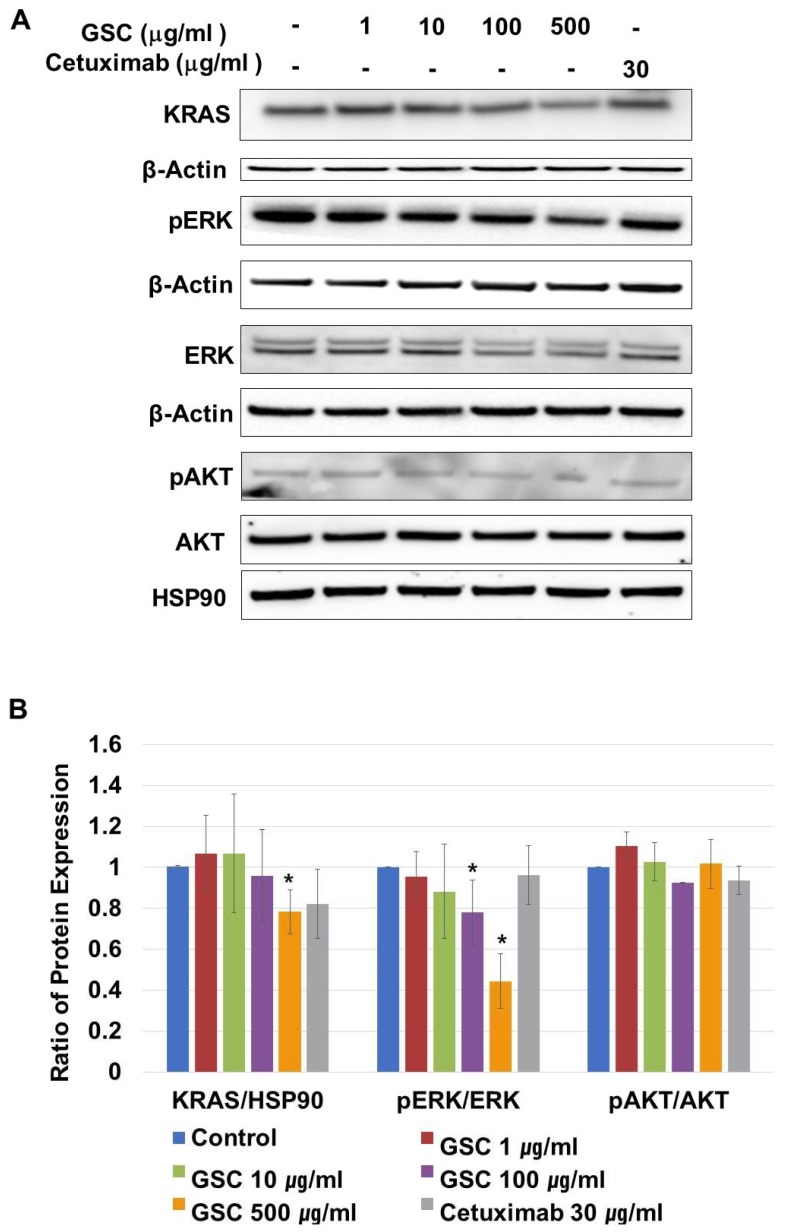
(**A**) Representative western blot images of KRAS, β-actin, phospho-p42/44 MAPK (phospho-ERK1/2), p42/44 MAPK (ERK1/2), phospho-AKT/AKT and HSP90 protein expression in SW480 cells treated with the GSC extract (0, 1, 10, 100, and 500 μg/mL). (**B**) The protein intensity was analyzed in triplicate. The ratios of KRAS/β-actin, phospho-p42/44/p42/44, and phospho-AKT/AKT were calculated. The ratios were compared with the no treatment group and significant differences are denoted by * (*p* < 0.05). The data are expressed as the mean ± standard error of the mean (SEM).

KRAS-mutated colorectal cancer is related to the RAS/ERK or PI3K/AKT pathways. On CRC progression, mutated KRAS proteins are produced continuously, which activate ERK-mediated transcriptional up-regulation. To determine if the suppression of SW480 growth by GSC was regulated by the RAS/ERK pathways, the cells were treated with GSC for 48 h. In addition, the expression levels of the KRAS, p-ERK, ERK, p-AKT, and AKT proteins were analyzed by Western blot. As shown in Figure 4A,B, the GSC treatment significantly suppressed KRAS expression and ERK phosphorylation. The GSC treatment decreased ERK phosphorylation significantly in a concentration-dependent manner. For example, the ratio of pERK/ERK in the control (1.00 ± 0.00) was reduced up to 0.44 ± 0.13 in the GSC 500 µg/mL-treated group. An alternative downstream of KRAS, which is the PI3K/AKT pathway, was also observed. The level of AKT phosphorylation was similar in all samples to that observed with the pAKT/AKT ratio. Based on the result, the GSC treatment suppressed colon cancer progression, which was mostly mediated by the RAS/ERK signaling pathway.

### 3.6. The GSC Extract Inhibits Tumor Growth in a Xenograft Colorectal Cancer Model 

**Figure 5:**
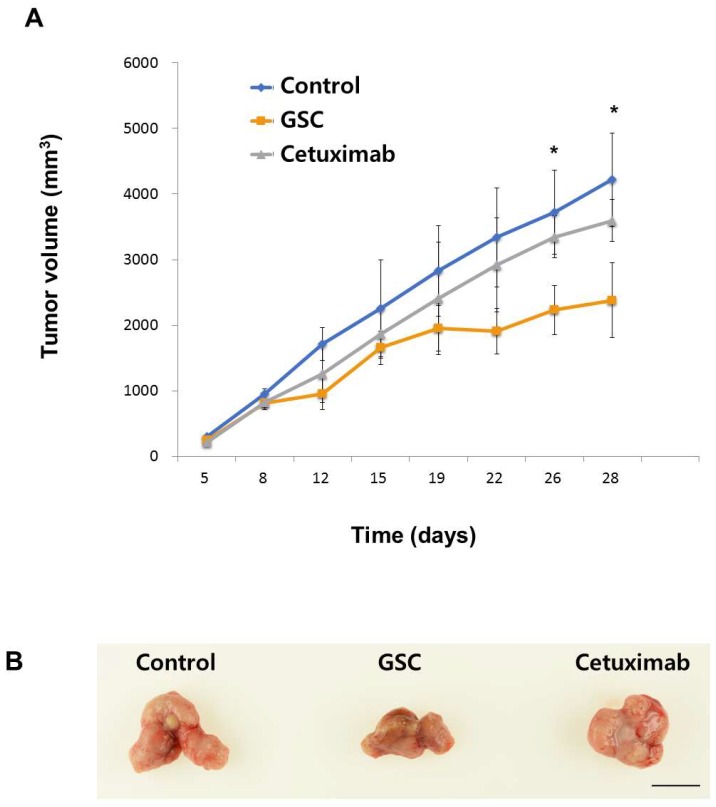
(**A**) Tumor volume changes in SW480 xenograft mice treated with the control (PBS), Cetuximab (10 mg/kg in PBS) and GSC extract (400 mg/kg/day) (*n* = 7). The tumor volumes were compared with those of the control treatment and a significant difference is denoted as * (*p* < 0.05). (**B**) Representative SW480 xenograft tumors were resected on day 26 showing the difference in tumor volume between the control or the Cetuximab treatment and the GSC extract treatment. Scale bar = 1 cm. The data are expressed as the mean ± standard error of the mean (SEM).

To investigate whether the GSC extract inhibits tumor progression, the SW480 xenograft animal model was generated. Tumor formation was induced by injecting SW480 cells and administering GSC daily. As shown in Figure 5, GSC suppressed tumor growth, which was confirmed by the significant decrease in tumor volume compared to the PBS control (PBS control vs. GSC extracts, mean tumor volume on day 26 = 3724 ± 714 mm^3^ vs. 2231 ± 581 mm^3^). No significant anti-cancer activity was observed in the Cetuximab control group. The body weights of the mice showed no significant changes in all groups, which suggests that GSC feeding is safe and well-tolerated.

## 4. Discussion

Colorectal cancer (CRC) is the third most prevalent cancer and is becoming the leading cause of cancer death [1,2]. In recent years, attempts to improve the survival rates in CRC have focused on innovative therapeutic methods including EGF-receptor antibody therapy such as Cetuximab [4]. On the other hand, 30% to 40% of patients with KRAS-mutated colorectal cancer do not benefit from Cetuximab therapy [5,6,7]. Natural compounds could be an attractive option for cancer therapy as a result of their safety and their applications. GSC has therapeutic activity for inflammatory conditions, allergies, and cancer. The current study showed that GSC treatment could suppress KRAS-driven colon cancer both *in vitro* and *in vivo*. GSC showed enhanced antitumor activity based on the results of cell proliferation and clonogenic potential in multiple KRAS-mutated colorectal cancer cells such as SW480 (KRAS^G12V^), LS513 (KRAS^G12D^) and HCT116 (KRAS^G13D^) (Figure 1). A less than 50% growth rate on KRAS-mutated CRC cell lines of SW480, LS513, and HCT116 was observed. In the KRAS^WT^ cell line of HT-29, Caco-2 and A549 cell proliferation was approximately 80%, which indicated that GSC is more effective on KRAS-mutated colon cancer (Figure 1). Clonogenic potential showed a similar tendency. KRAS-mutated cell lines showed significant suppression on the clonogenic potential (Figure 2). KRAS-mutated cell death was from the increasing apoptosis with GSC treatment (Figure 3). The RAS/ERK pathway study suggested that GSC could show an antitumor effect through the modulation of the ERK pathway by altering the phosphorylation of ERK (Figure 4). In a human tumor growth model, the growth of KRAS mutant xenograft tumors was suppressed significantly by the GSC treatment (Figure 5).

The RAS/ERK signaling pathway and its downstream factors are recognized as key regulators for cellular behaviors including cell survival, proliferation, invasion, and migration [3]. During CRC progression, mutant RAS constitutively activates ERK phosphorylation. Therefore, this study examined their changes upon the GSC treatment and found decreased KRAS expression and phosphorylation of ERK proteins in SW480 cells (Figure 3). 

*Cordyceps militaris* on germinated soybeans contains several bioactive compounds. In particular, isoflavone methyl-glycosides, cordycepin, adenine, and adenosine have been identified [19,20]. Four isoflavone methyl-glycosides including daidzein 7-*O*-b-d-glucoside 4″-*O*-methylate, glycitein 7-*O*-b-d-glucoside 4″-*O*-methylate, genistein 7-*O*-b-d-glucoside 4″-*O*-methylate, and genistein 4′-*O*-b-d-glucoside 4″-*O*-methylatewere identified to exhibit anti-allergic activity in antigen-stimulated mast cells by suppressing ERK phosphorylation [19,21]. Genistein induced apoptosis on leukemia and colon cancer and inhibited breast cancer cell proliferation [25,26]. Cordycepin (a nucleoside derivative), which is a major active component of GSC, induces apoptosis in melanoma and lung carcinoma [27,28]. Furthermore, adenosine from GSC suppressed the growth of human gastric cancer cells significantly and the adenosine receptor 1 suppressed colon cancer proliferation [29]. The anti-tumor effects of GSC might come from the above functional compounds within the GSC.

In our current study, GSC contains relatively low Met 0.55 mg/g and high Arg 1.42 mg/g crude (Table 2). Several studies have examined the effects of individual amino acids such as Met, Ser, Gly, and Arg on cancer progression. Met restriction was reported to inhibit cancer cell growth and to extend a healthy lifespan in several animal studies [30]. Met-depletion in cancer cells was reported to lead to cell cycle arrest. Deprivation of dietary Ser and Gly inhibits the growth of tumors by limiting nucleotide biosynthesis [31]. When Arg was provided at 1 mmol/L, it suppressed colon cancer by lowering the angiogenesis-related growth factors [32,33]. Relatively low Met 0.55 mg/g and high Arg 1.42 mg/g crude might play roles in suppressing colon cancer.

## 5. Conclusions

In conclusion, these observations suggest that GSC suppressed CRC progression with inhibitory effects, stopping the essential steps of tumor growth. GSC may be a powerful candidate for the development of a preventive agent for cancer growth. To the best of the authors’ knowledge, this is the first study reporting that a GSC treatment suppresses KRAS-mutated colon cancer through the ERK-dependent pathways. Given the non-toxic nature of these natural food substances and their beneficial activities, these results provide support for the incorporation of the GSC extract into therapeutic regimens for colorectal cancer.

## Figures and Tables

**Table 1:** Proximate composition of *Cordyceps militaris* on germinated soybeans (GSC).

Compound (% of Lyophilisate)	
Crude protein	55
Crude fat	1
Crude ash	13

**Table 2:** Amino acid composition of GSC (mg/g).

Amino Acids	Essential Amino Acids (EAA)
Isoleucine	0.76 ± 0.00
Leucine	2.53 ± 0.00
Lysine	1.94 ± 0.17
Methionine	0.55 ± 0.00
Phenylalanine	1.29 ± 0.04
Threonine	3.06 ± 0.09
Valine	2.21 ± 0.06
Tryptophan	0.44 ± 0.07
	**Half-Essential Amino Acids (HEAA)**
Arginine	1.42 ± 0.02
Histidine	0.76 ± 0.17
	**Non-Essential Amino Acids (NEAA)**
Cysteine	0.27 ± 0.00
Aspartic acid	3.16 ± 0.04
Glycine	1.81 ± 0.03
Glutamic acid	16.40 ± 0.32
Alanine	4.11 ± 0.08
Serine	3.51 ± 0.09
Proline	1.18 ± 0.06
Tyrosine	1.50 ± 0.06
	**Amino Acid Groups and Ratios**
Total amino acids (TAA)	46.9 ± 1.3
Essential amino acids (EAA)	12.78 ± 0.43
Half-essential amino acids (HEAA)	2.18 ± 0.19
Non-essential amino acids (NEAA)	31.94 ± 0.68
Delicious amino acids (DAA)	25.48 ± 0.47
EAA/TAA	0.27
EAA/NEAA	0.40
DAA/TAA	0.54
